# Cryo-EM of the ATP11C flippase reconstituted in Nanodiscs shows a distended phospholipid bilayer inner membrane around transmembrane helix 2

**DOI:** 10.1016/j.jbc.2021.101498

**Published:** 2021-12-17

**Authors:** Hanayo Nakanishi, Kenichi Hayashida, Tomohiro Nishizawa, Atsunori Oshima, Kazuhiro Abe

**Affiliations:** 1 Cellular and Structural Physiology Institute, Nagoya University, Nagoya, Japan; 2 Graduate School of Medical Life Science, Yokohama City University, Yokohama, Japan; 3 Graduate School of Pharmaceutical Sciences, Nagoya University, Nagoya, Japan; 4 Institute for Glyco-core Research (iGCORE), Nagoya University, Nagoya, Japan

**Keywords:** P-type ATPases, P4-ATPases, active transport, apoptosis, cryo-EM, flippase, membrane, Nanodisc, cryo-EM, cryo-electron microscopy, DOPC, dioleoylphosphatidylcholine, DOPS, dioleoylphosphatidylserine, MβCD, methyl-β-cyclodextrin, MSP, membrane scaffolding protein, PtdCho, phosphatidylcholine, PtdEtn, phosphatidylethanolamine, PtdSer, phosphatidylserine, SEC, size-exclusion chromatography

## Abstract

ATP11C is a member of the P4-ATPase flippase family that mediates translocation of phosphatidylserine (PtdSer) across the lipid bilayer. In order to characterize the structure and function of ATP11C in a model natural lipid environment, we revisited and optimized a quick procedure for reconstituting ATP11C into Nanodiscs using methyl-β-cyclodextrin as a reagent for the detergent removal. ATP11C was efficiently reconstituted with the endogenous lipid, or the mixture of endogenous lipid and synthetic dioleoylphosphatidylcholine (DOPC)/dioleoylphosphatidylserine (DOPS), all of which retained the ATPase activity. We obtained 3.4 Å and 3.9 Å structures using single-particle cryo-electron microscopy (cryo-EM) of AlF- and BeF-stabilized ATP11C transport intermediates, respectively, in a bilayer containing DOPS. We show that the latter exhibited a distended inner membrane around ATP11C transmembrane helix 2, possibly reflecting the perturbation needed for phospholipid release to the lipid bilayer. Our structures of ATP11C in the lipid membrane indicate that the membrane boundary varies upon conformational changes of the enzyme and is no longer flat around the protein, a change that likely contributes to phospholipid translocation across the membrane leaflets.

In the plasma membrane of eukaryotic cells, phosphatidylserine (PtdSer) and phosphatidylethanolamine (PtdEtn) are localized in the inner leaflet (cytosolic side), whereas phosphatidylcholine (PtdCho) and sphingomyelin (SM) are abundant in the outer leaflet (exoplasmic side) (1, 2). This asymmetric distribution of phospholipids is a prerequisite for vesicular trafficking (3), cell signaling, and other cellular processes (4). In particular, controlled exposure of PtdSer to the cell surface is essential in physiological processes such as blood coagulation and apoptosis (5, 6, 7). Three classes of membrane transporters mediate phospholipid translocation across the lipid bilayer; scramblases, flippases, and floppases (8, 9, 10, 11). Scramblases mediate ATP-independent, nonselective, and bidirectional movement of phospholipids across the bilayer when they are activated by Ca^2+^ or caspases digestion (5, 12). Flippases, P4-ATPases, transport a specific lipid from the outer to the inner leaflet (13). Floppases, ATP-binding cassette transporters, translocate specific lipids mainly in the opposite direction to that mediated by flippases (14).

The P-type ATPase superfamily mediates ATP-dependent transport of substrates across the membrane. P-type ATPases are classified into five families according to their primary structure: P1 ATPases are heavy metal and potassium transporters; P2-ATPases are cation pumps; P3-ATPases are H^+^-pumps in plants; P4-ATPases are lipid flippases; P5-ATPases mediate polyamine (15) and short-helix uptake (16). In contrast to other family members, P4- and P5-ATPases are known for transporting large substrates. P4-ATPases are widely distributed in eukaryotes, with the human genome having 14 genes encoding P4-ATPases. Their dysfunction causes various metabolic and neurological disorders. Of all the P4-ATPases, ATP11C is of remarkable physiological importance. This is because ATP11C is one of the primary PtdSer flippases in the plasma membrane, and its caspase-dependent degradative inactivation upon apoptosis is required to expose PtdSer as an “eat me” signal of a dead cell (17).

Most P4-ATPases function as a heterodimer consisting of a P4-ATPase as a catalytic subunit and a CDC50 family protein as an accessory subunit. The overall fold of P4-ATPases is similar to the well-studied P2-type ATPases (18, 19, 20). The catalytic subunit comprises a cytosolic region responsible for ATPase hydrolysis and a transmembrane region. The cytosolic region consists of three functional domains; the nucleotide (N) domain binds ATP; the phosphorylation (P) domain contains the aspartate residue in the invariant DKTG motif that undergoes autophosphorylation; the actuator (A) domain has the DGES (TGES in P2-type ATPases) motif that expedites dephosphorylation. The transmembrane region has ten transmembrane helices (TM1–10) that serve as the phospholipid translocation pathway. The pathway is a hydrophilic groove penetrating the bilayer, appears transiently during the transport cycle, and accommodates a polar head group of the specific phospholipid leaving the acyl chains in the membrane core. The P4-ATPases transport their substrate through a cyclic transition of four cornerstone states, E1–E1P–E2P–E2, according to the Post–Albers-type reaction scheme (13, 21).

We previously reported a crystal structure (12) and cryo-EM structures (13) of a detergent-solubilized ATP11C-CDC50A complex together with complementary biochemical analyses, which shed light on the molecular mechanism of its PtdSer transport (22, 23). Apart from ours, several other P4-ATPase structures have been determined, including *S. cerevisiae* Drs2-Cdc50 (24, 25), Dnf1-Lem3 (26), Dnf2–Lem3 (26), *C. thermophilum* Dnf1-Cdc50 (27), and human ATP8A1-CDC50A (28). Despite the abundance of reported structures in different states of the transport cycle, fundamental questions remain, including how phospholipids are incorporated into and released from the transport conduit, in the face of the energetically favorable membrane bilayer environment. Further insight into its transport mechanism is likely to come from structural and functional studies in a controlled lipid bilayer environment. A Nanodisc is a synthetic model membrane system that enables structural and functional analysis of membrane proteins in a natural context. It is composed of a phospholipid bilayer with the hydrophobic edge screened by two amphipathic proteins called membrane scaffolding proteins (MSP) and aligns in double belt formation (29, 30, 31). Reconstitution of membrane proteins into Nanodiscs is achieved by detergent removal from mixed micelles of the protein samples, usually by dialysis or the addition of polystyrene beads (*e.g.*, Bio-beads). However, complete detergent removal by these procedures requires many hours often including overnight incubation, and it is unsuitable for those proteins with susceptibility to detergent perturbation, which causes low yields and even denaturation during reconstitution; hence, the need for a quick and quantitative approach. We then revisited a previously reported Nanodisc reconstruction procedure using β-cyclodextrin derivatives for detergent removal in the preparation of proteoliposomes (32) and lipid Nanodiscs (33, 34). Using this approach, we achieved ATP11C reconstitution into lipid Nanodiscs in just 15 min without significant loss of protein, allowing us to investigate its ATPase activity as well as structural properties by cryo-EM analysis in a model native lipid bilayer. The ATP11C-Nanodisc structure of a crucial transport intermediate revealed a protruded membrane surface with transmembrane helices, which is likely involved in the process of PtdSer release to the inner leaflet of the plasma membrane.

## Results

### Empty Nanodisc assembly

**Figure 1:**
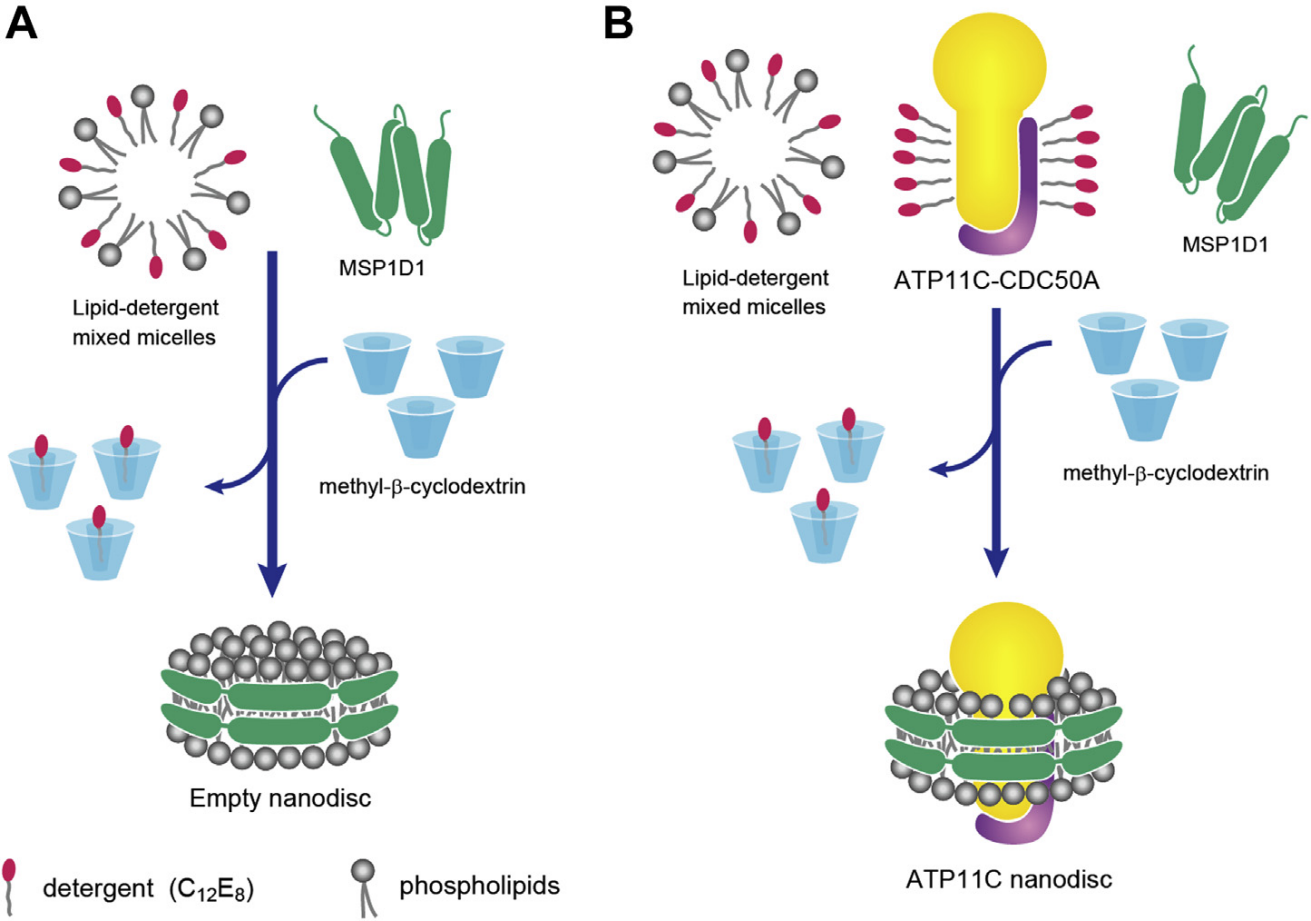
**Schematic representations of the Nanodisc assembly using MβCD.***A*, empty-Nanodiscs were generated with phospholipids solubilized in 2% C_12_E_8_ and MSP1D1 by detergent removal using MβCD. *B*, the affinity-purified ATP11C-CDC50A complex was reconstituted in phospholipid Nanodiscs as with the empty-Nanodisc assembly.

Nanodisc assembly is likely driven by the detergent removal, so we tried to determine the key parameters for reconstitution by making “empty-Nanodiscs” in a simplified system (Fig. 1*A*), using only MSP, phospholipid, and detergent, notably excluding target protein ATP11C. In this study, we use MSP1D1 and octaethyleneglycol monododecylether (C_12_E_8_) as membrane scaffold and detergent, respectively. MSP1D1 has been utilized for Nanodisc reconstitution of other P-type ATPases such as SERCA and Na^+^,K^+^-ATPase, and it is expected to be suitable for ATP11C too because of its similar size. The detergent C_12_E_8_ has also been used for many P-type ATPases, and indeed ATP11C has been successfully purified and crystallized using this detergent (22). We employ methyl-β-cyclodextrin (MβCD), instead of the widely used polystyrene beads such as Bio-beads, as a detergent removal agent (35). MβCD is a highly water-soluble β-cyclodextrin derivative and has already been utilized for proteoliposome and Nanodisc reconstitution.

**Figure 2:**
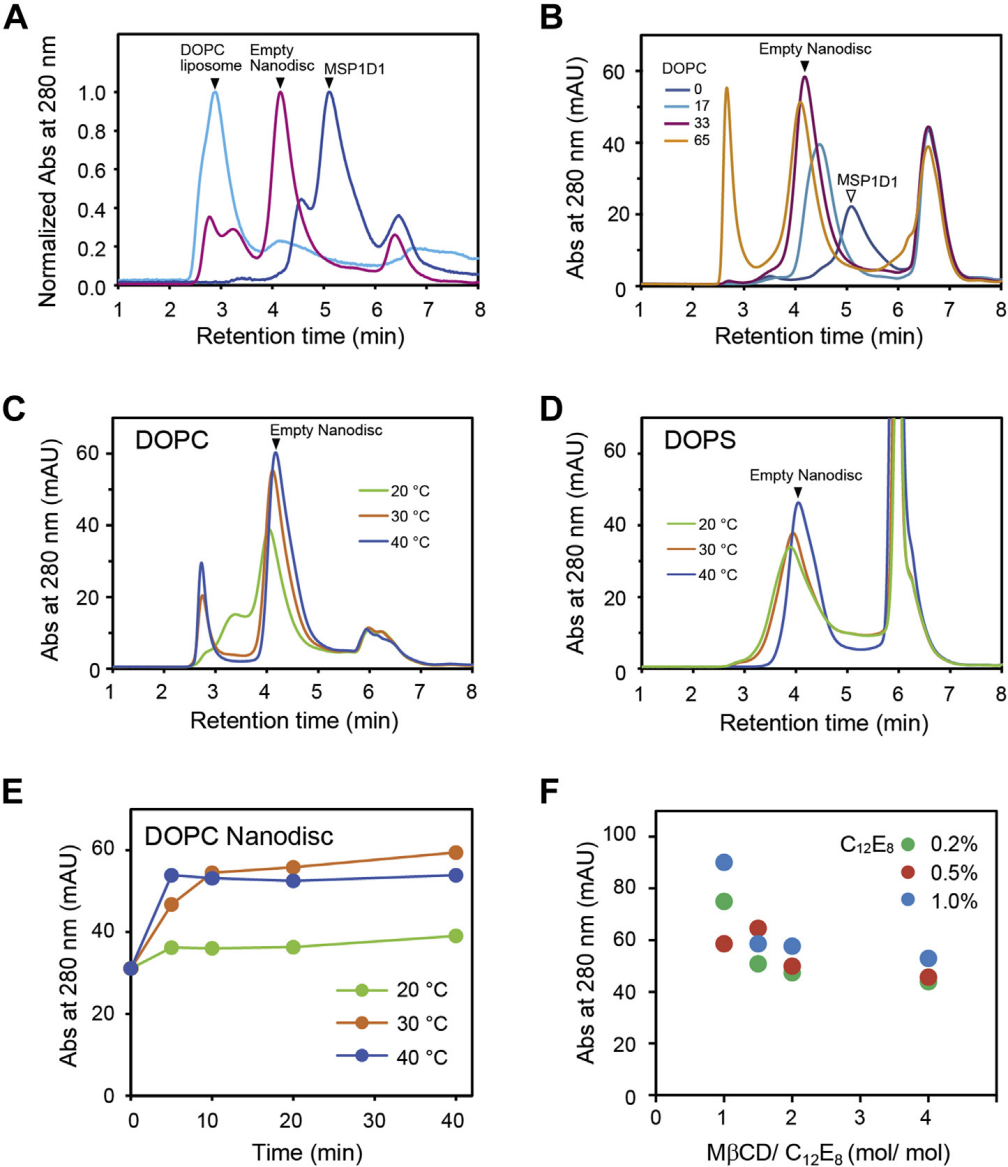
**Empty-Nanodiscs assembly.** Empty-Nanodiscs were generated with phospholipids and MSP1D using MβCD. *A*, peaks of DOPC liposomes (*light blue*), empty DOPC Nanodiscs (*magenta*), and free MSP1D1 (*blue*) were identified in the SEC profiles. The values of absorbance at 280 nm were normalized by setting the maximum values of each peak as 1.0 (DOPC: 8.2 mAU, empty Nanodisc: 26 mAU, MSP1D1: 9.2 mAU). *B*, the empty-Nanodiscs were assembled with DOPC at molar ratio MSP1D1:DOPC = 1:0, 1:17, 1:33, 1:65. *C* and *D*, the temperature effects were investigated for the empty-Nanodisc formed with DOPC (*C*) or DOPS (*D*) during detergent removal at MSP1D1:phospholipids = 1:33. Incubation after adding MβCD was carried out for 10 min at the indicated temperature. *E*, the time course of empty-Nanodisc production was examined at the indicated temperature after adding MβCD at MSP1D1:DOPC = 1:33. The peak values at 4.2 min were plotted against incubation time. *F*, the ratio of MβCD:C_12_E_8_ was varied at MSP1D1:DOPC = 1:33 with 0.2%, 0.5% and 1.0% of C_12_E_8_. Incubation after adding MβCD was for 10 min at 30 °C. The peak values at 4.2 min were plotted against the molar ratio of MβCD to C_12_E_8_.

Small-scale titration experiments (100 μl) for empty-Nanodisc reconstitution were conducted by mixing MSP1D1, phospholipid, and C_12_E_8_ in a controlled molar ratio, followed by addition of MβCD to remove the detergent. The incubation time and temperature also proved to be important variables. Buffers did not contain glycerol because it interferes with Nanodisc assembly (30). Resulting insoluble material was removed through a 0.22 μm filter, and 40 μl of the reactant was subjected to analytical scale size-exclusion chromatography (SEC) using a detergent-free SEC buffer at room temperature. The quantitative detection of products such as liposomes (2.9 min), empty-Nanodiscs (4.2 min) and the monomeric form of MSP1D1 proteins (5.2 min) can be achieved according to their apparent molecular weight (Fig. 2*A*). Note, it was essential to monitor the SEC profiles by absorbance at 280 nm rather than tryptophan fluorescence (excitation 280 nm, emission 320 nm) for the quantitative detection of the products. Although tryptophan fluorescence is highly sensitive, MSP contains Trp residues whose fluorescence quantum yield likely changes according to their microenvironment (temperature, hydrophobicity, and others), giving variable fluorescence intensities.

We next titrated an appropriate amount of phospholipid (here we used DOPC) for empty-Nanodisc formation in molar ratios MSP1D1: DOPC = 1:0, 1:17, 1:33, 1:65 (Fig. 2*B*) according to a previous report (30). Excess DOPC (1:65) produced a liposome that peaked at 2.7 min, and the empty-Nanodisc was retained at 4.2 min. On the other hand, fewer DOPC (1:17) produced a broad peak at 4.4 min and attenuated the empty-Nanodisc yield, indicating that the amount of phospholipid is an important factor for successful Nanodisc formation. Thus, we selected the ratio of MSP1D1: DOPC =1:33, and this proportion is in good agreement with the number of phospholipids (approximately 70 molecules) expected in a single Nanodisc formed with two MSP molecules (30).

In the fixed molar ratio of MSP1D1: DOPC = 1:33 determined above, we examined the effect of incubation temperature after MβCD addition (Fig. 2*C*, samples for 10 min incubation). As the temperature increased, the retention time of the empty-Nanodisc shifted from 4.0 min to 4.2 min, and the peak became sharper and higher. Similar results were obtained for the DOPS Nanodisc (Fig. 2*D*). The time course for DOPC Nanodisc formation at different temperatures (Fig. 2*E*) revealed that the yields reach a plateau approximately 10 min after MβCD addition at 30 °C and 40 °C, the latter temperature seemingly a bit more effective. A long incubation at 20 °C did not improve yield over those at 30 °C and 40 °C. Considering that similar time courses were obtained at 30 °C and 40 °C and the potential risk of protein denaturation, we chose 10 min incubation at 30 °C as the standard protocol for detergent removal by MβCD addition.

Finally, we evaluated the effect of the molar ratio of MβCD and detergent for the empty-Nanodisc formation in the presence of 0.2 to 1.0% (4–19 mM) C_12_E_8_ in the fixed condition of MSP1D1:DOPC = 1:33 and 10 min incubation at 30 °C (Fig. 2*F*). The trends shown in Figure 2*F* indicate that excess MβCD drops the yield, which is likely due to the removal of phospholipid over detergent, at least in the case of empty-Nanodisc assembly. The result is roughly consistent with the appropriate MβCD ratio to detergent 1 to 1.5 to prepare proteoliposomes (32). Thus, we chose MβCD:C_12_E_8_ = 1:1, which is logical because MβCD binds stoichiometric amounts of C_12_E_8_ (Fig. 1).

### ATP11C-Nanodisc assembly

**Figure 3:**
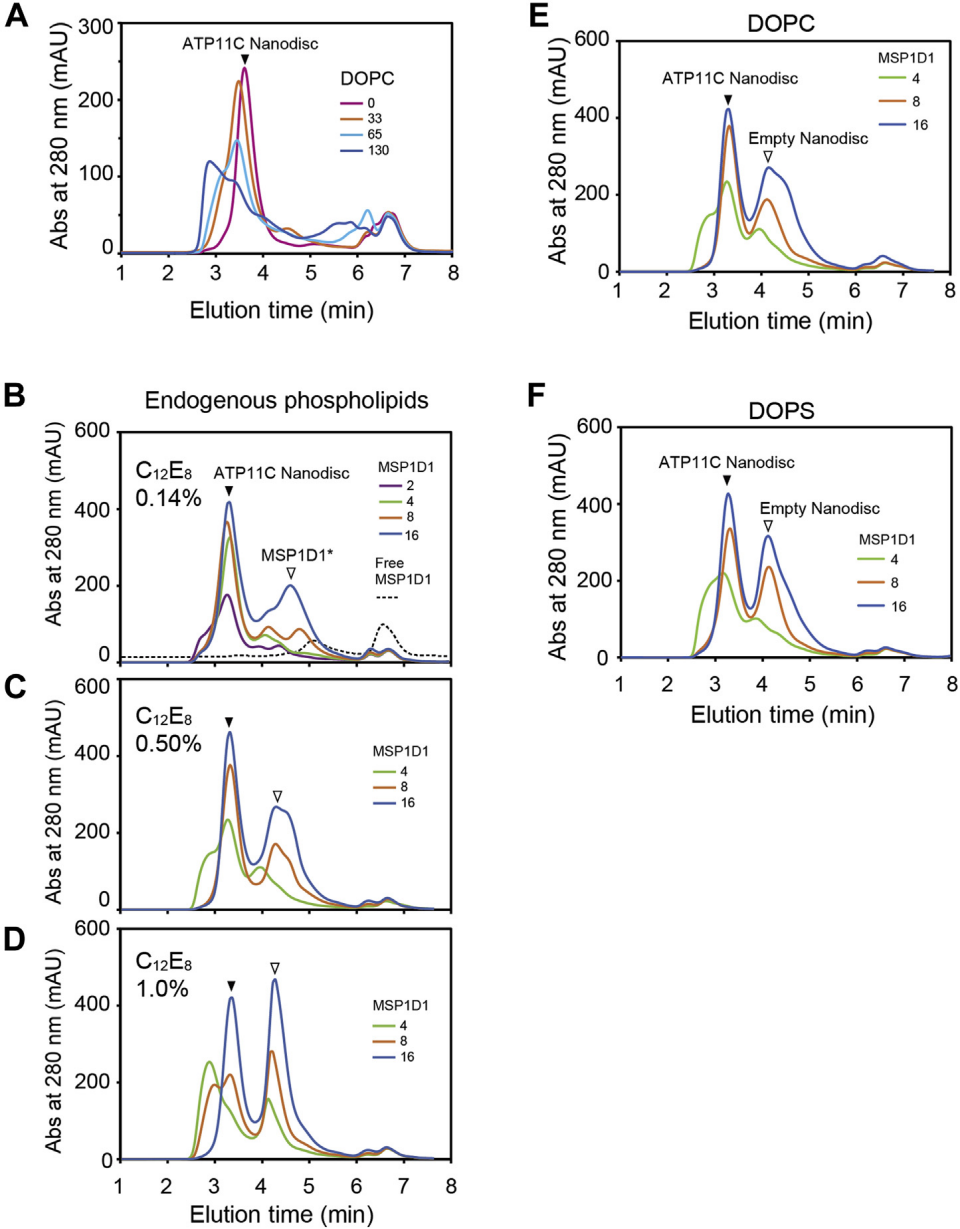
**ATP11C-Nanodisc assembly.** The affinity-purified ATP11C was incorporated into Nanodiscs using MβCD. *A*, the ratios ATP11C:DOPS, 1:0, 1:33, 1:65, 1:130 were tested, fixing the ratio of ATP11C:MSP1D1 = 1:2. *B*–*D*, the ATP11C-Nanodiscs were generated with endogenous phospholipids and MSP1D1. The molar ratios ATP11C:MSP1D1 = 1:2, 1:4, 1:8, 1:16 were examined in the presence of C_12_E_8_ at concentrations 0.14% (*B*), 0.50% (*C*), 1.0 (*D*). *E* and *F*, ATP11C was incorporated into Nanodiscs with DOPC (*E*) or DOPS (*F*). The ratio ATP11C:phospholipids was fixed at 1:33 in the presence of 0.5% C_12_E_8_. The ratios ATP11C:MSP1D1, 1:4, 1:8, 1:16 were tested.

In contrast to the simplified empty-Nanodisc system described above, the experimental concentration of detergent when the purified ATP11C-CDC50A complex (hereafter ATP11C) is included is not easily determined, but can be estimated from the amount of detergent carried over. The ATP11C samples (0.5 mg/ml × 10 ml), eluted from the affinity-purified resin equilibrated with a buffer containing 0.03% C_12_E_8_, were concentrated to approximately 5 mg/ml by the ultrafiltration membranes (MWCO 50,000 Da) through which the C_12_E_8_ micelles rarely pass. Consequently, the concentration of C_12_E_8_ in the purified ATP11C sample can be assumed to be approximately 0.3%. We evaluated ATP11C-Nanodisc assembly in 100 μl by adding MβCD to the mixture at a molar ratio of MβCD: C_12_E_8_ = 1:1, followed by the 10 min incubation at 30 °C unless otherwise noted (Fig. 3).

Since a Nanodisc assembles with two MSP molecules that surround a membrane disc with the reconstituted membrane protein (Fig. 1*B*), we fixed the molar ratio of ATP11C: MSP1D1 = 1:2 to avoid empty-Nanodisc production (29) and tested various concentrations of DOPC at the molar ratios of ATP11C:DOPC = 1:0, 1:33, 1:65, 1:130 (corresponding to the molar ratio of MSP1D1:DOPC = 1:0, 1:16, 1:33, 1:66, respectively) (Fig. 3*A*). In contrast to the case for empty-Nanodisc (Fig. 2*B*), ATP11C-reconstituted Nanodisc unexpectedly formed in the absence of DOPC, and the yield is highest when phospholipid is not added (Fig. 3*A*). The reconstitution of ATP11C is evident as the peak (3.3 min) obtained with the ATP11C sample is significantly shifted to the higher molecular weight side compared with that of empty-Nanodisc (4.2 min). Determination of the amount of phospholipid in the purified ATP11C sample indicated that approximately 49 phospholipid molecules are capsulated in a single ATP11C-Nanodisc, and 92 phospholipid molecules are contained in a single affinity-purified ATP11C molecule, despite extensive washing with the buffer containing 0.03% C_12_E_8_ during the purification step (Table 1). These results indicate that the affinity-purified ATP11C contains endogenous phospholipids carried over from the cell membrane, the amount of which is high enough to constitute an ATP11C-Nanodisc without external phospholipid addition. ATP11C-Nanodisc formed with externally added DOPC and with DOPS (3.3 min) gave almost the same molecular weight.

**Table 1:** Determination of total phosphorus in the sample

Phospholipids	Nanodiscs		C_12_E_8_ micelles
	Empty	ATP11C	ATP11C
Endogenous lipids	-	49	92
DOPC	98	56	-
DOPS	31	58	-

The amount of phosphorus derived from the phospholipids in the samples is indicated as moles/mole of ATP11C. Data represent the mean of triplicate measurements.

For the structural and functional analysis of ATP11C-Nanodisc, at least sub-mg samples are required. Then the concentration of the detergent carried over in the purified ATP11C sample can be relatively high. Therefore, we evaluated the effect of detergent concentrations on Nanodisc formation. When we set the concentration of ATP11C to 1.5 mg/ml without external phospholipid addition, the minimal possible concentration of C_12_E_8_ is 0.14% in the reconstitution solution. In this condition, ATP11C:MSP1D1 = 1:8 M ratios gave a higher yield for ATP11C-Nanodisc production compared with those for lower concentrations of MSP1D1 (Fig. 3*B*), and the yield is essentially saturated. Although further addition of MSP1D1 (1:16) apparently increased the yield, this is likely due to contamination from excess MSP1D1 separated in a monomeric form (Fig. 3*B*). The more C_12_E_8_ present, the more MSP1D1 required for ATP11C-Nanodisc formation with its endogenous lipids (Fig. 3, *B*–*D*), and this is also the case for ATP11C-Nanodisc formation with exogenously added DOPC or DOPS in the presence of 0.5% C_12_E_8_ (Fig. 2, *E* and *F*). Accordingly, although a lower C_12_E_8_ concentration is favorable for ATP11C-Nanodisc reconstruction, excess MSP1D1 (up to 16 times higher molar ratio per ATP11C) is beneficial when the concentration of C_12_E_8_ is high in the purified sample.

**Figure 4:**
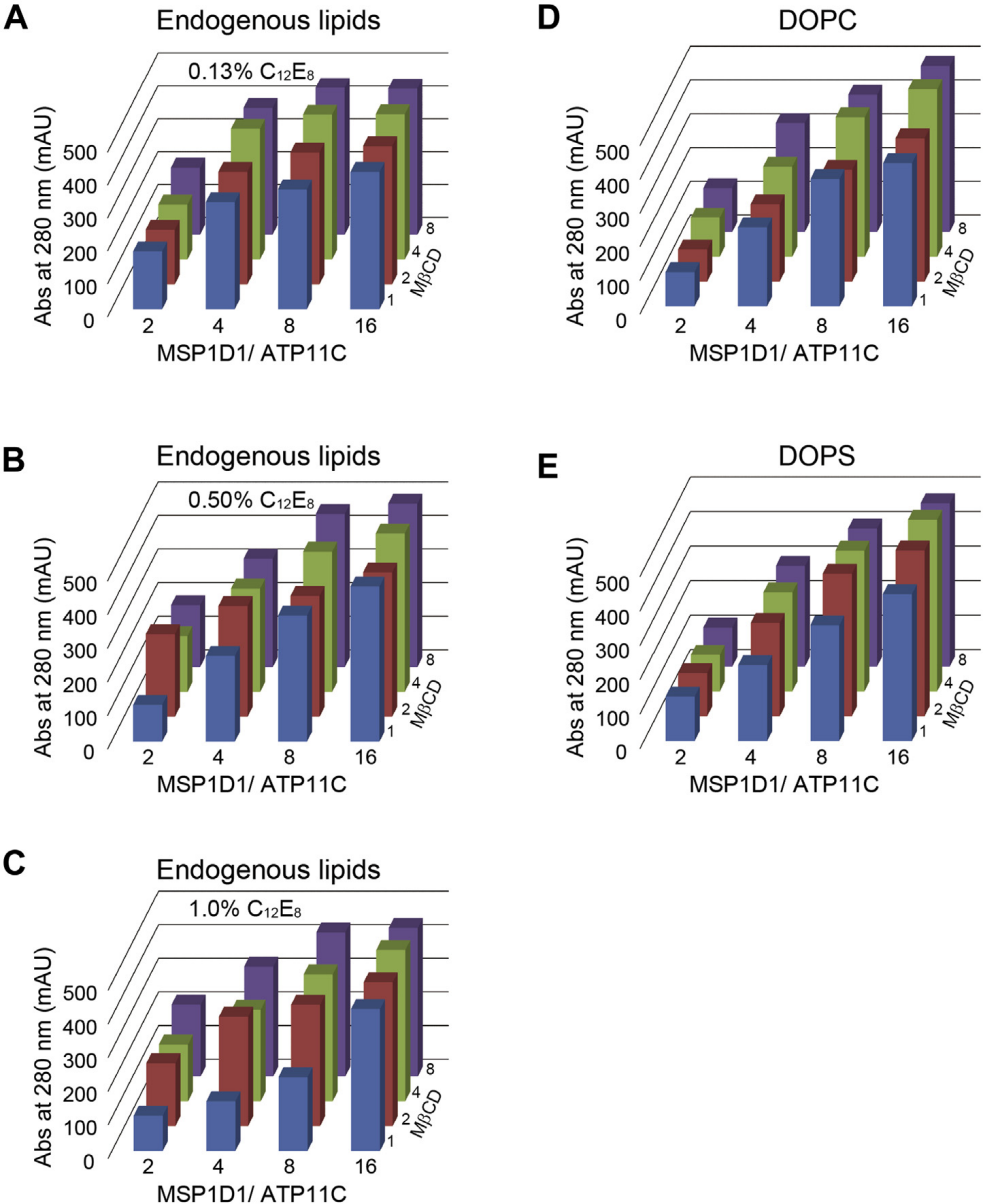
**Effect of the molar ratios of MSP1D1 to ATP11C and those of MβCD to C**_**12**_**E**_**8**_**on ATP11C-Nanodisc assembly.** The affinity-purified ATP11C was incorporated into Nanodiscs with different molar ratios MSP1D1 to ATP11C (x-axis) and MβCD to C_12_E_8_ (y-axis). *A*–*C*, Nanodiscs were assembled with endogenous lipid in the presence of C_12_E_8_, 0.13%, 0.5%, 1.0%. Nanodiscs with DOPC (*D*) and DOPS (*E*) were constructed at 0.5% C_12_E_8_. One bar in the graphs represents the peak height of the ATP11C-Nanodiscs eluted at 3.3 min under each condition.

We also conducted systematic comparisons of the relationship between MSP1D1 and MβCD added to the different concentrations of C_12_E_8_ for ATP11C-Nanodisc formation with endogenous lipids (Fig. 4, *A*–*C*) and also with exogenously added DOPC (Fig. 4*D*) or DOPS (Fig. 4*E*). An increase in MβCD molar ratio marginally improved yields, but the effect was limited compared with the cases in the presence of excess MSP1D1. For example, when we compare the yield of ATP11C-Nanodisc in the presence of MSP1D1/ATP11C molar ratio of 4 and 16 with various concentrations of MβCD in 1.0% C_12_E_8_ (Fig. 4*C*), excess MβCD improves yield in the presence of molar ratio 4 MSP1D1/ATP11C (from ∼150 mAU in 1 MβCD to ∼300 mAU in 8 MβCD). The low yield in the presence of low concentrations of MβCD is likely due to insufficient detergent removal, and the yield can be recovered to a certain extent with more MβCD. On the other hand, the yield with molar ratio 16 MSP1D1/ATP11C gives much higher values at all MβCD concentrations tested (∼400 mAU), compared with those with molar ratio 4 MSP1D1 (∼300 mAU in 8MβCD). These data indicate that excess MSP1D1 helps ATP11C-Nanodisc formation especially in the presence of a high concentration of C_12_E_8_. However, excess MSP1D1 simultaneously increases empty-Nanodisc production (Fig. 3), which may interfere with cryo-EM analysis, and therefore careful titration of MSP1D1 concentration would be crucial.

### Preparative assembly of ATP11C Nanodisc

**Figure 5:**
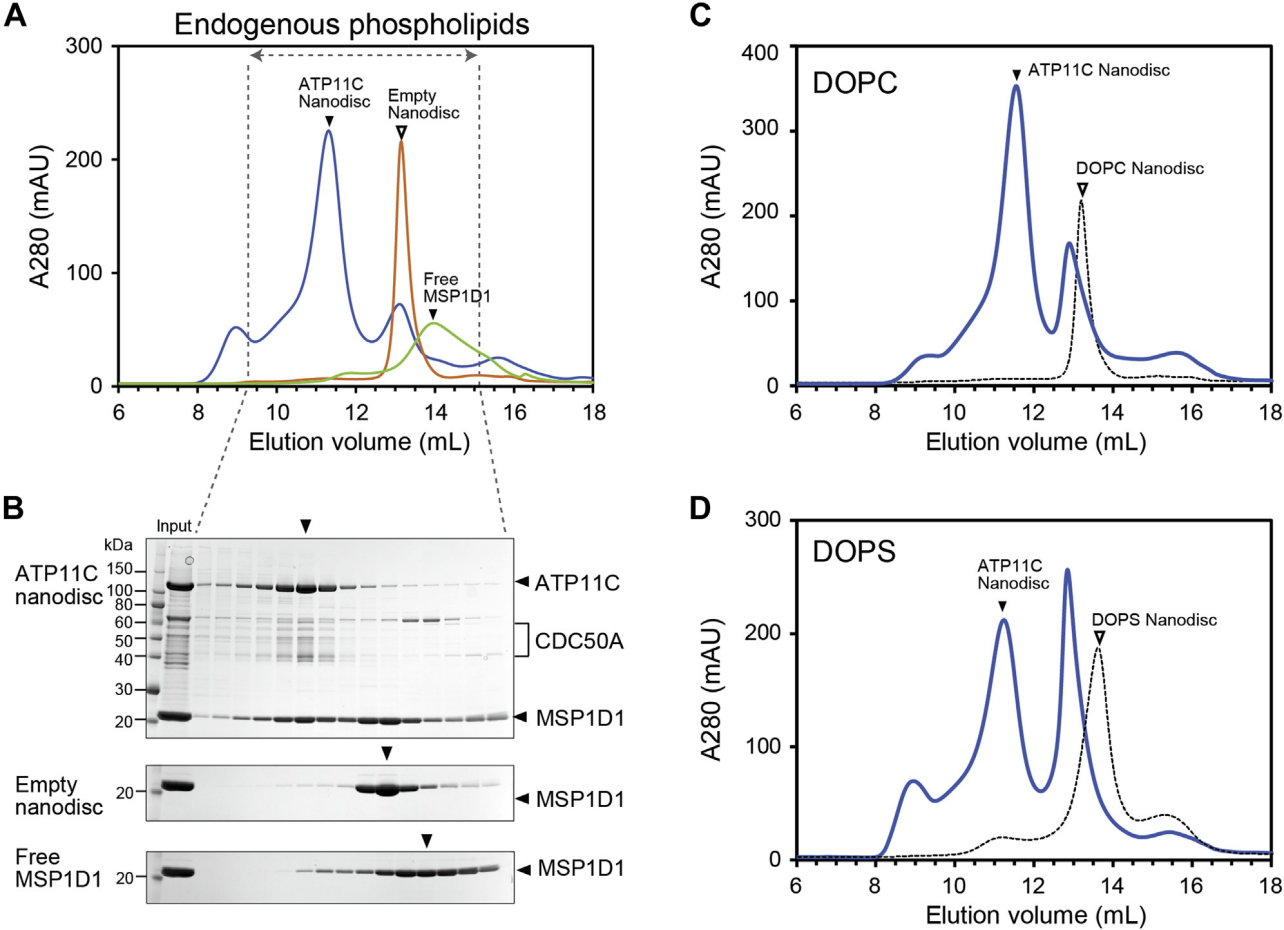
**Preparative assembly of the ATP11C Nanodiscs.***A*, ATP11C-Nanodiscs were constructed with endogenous phospholipid. The mixture was incubated for 15 min at 30 °C after adding MβCD, and separated by SEC using Superdex 200 Increase 10/300 at 4 °C. The ratio ATP11C:MSP1D1 was set to 1:4 in the presence of 0.2% C_12_E_8_. Empty-Nanodiscs assembled with DOPC and free MSP1D1 were also separated by SEC. *B*, aliquots of the SCE fractions were analyzed by SDS-PAGE. *C* and *D*, ATP11C-Nanodiscs were assembled with DOPC (*C*) or DOPS (*D*). The ratio ATP11C:MSP1D1 was set to 1:8 in the presence of 0.5% C_12_E_8_.

ATP11C-Nanodisc reconstitution on a preparative scale was necessary for structural and functional studies in a controlled lipid environment. As seen above, a critical factor is the detergent concentration, which is carried over from the purified and concentrated ATP11C sample. We calculated the concentration of C_12_E_8_ to be 0.25% (4.6 mM) in the reconstitution mixture. Accordingly, we set the molar ratio of ATP11C:MSP1D1 = 1:8 in the absence of exogenous phospholipid. The process was started by adding an aliquot of 100 mM MβCD in the molar ratio of MβCD:C_12_E_8_ = 1:1 to the 1 ml reaction mixture and incubation for 15 min at 30 °C, followed by centrifugation at 100,000*g* for 20 min at 4 °C to remove insoluble material. Recovery of protein in the supernatant determined by the absorbance at 280 nm was more than 95%, indicating almost no loss of ATP11C. The supernatant was separated by SEC using Superdex 200 Increase 10/300 GL (Fig. 5*A*), and the fractions were analyzed by SDS-PAGE (Fig. 5*B*). The peak fraction (in 11.3 ml elution volume) contained ATP11C, CDC50A (smeary band due to glycosylation), and MSP1D1, and it was well separated from both the empty Nanodisc (13.3 ml) and the monomeric MSP1D1 (14.0 ml), demonstrating successful formation of ATP11C-Nanodisc with endogenous lipids.

The ATP11C-Nanodisc with exogenous DOPC (Fig. 5*C*) or DOPS (Fig. 5*D*) was also generated by setting the molar ratio of ATP11C:MSP1D1:DOPC 1:16:33 in 0.5% C_12_E_8_. As is the case for ATP11C-Nanodisc formed with endogenous lipids (Fig. 5*A*), ATP11C-Nanodisc with DOPC (11.3 ml) or DOPS (11.6 ml) was successfully separated from the empty-Nanodisc fractions. For DOPC, the elution volume of empty-Nanodisc in the ATP11C-containing sample overlapped with the peak corresponding to the empty-Nanodisc produced with pure DOPC (13.2 ml). However, pure DOPS empty-Nanodisc eluted in the smaller molecular weight fractions (13.7 ml) compared with pure DOPC empty-Nanodisc and the empty-Nanodisc produced in the presence of ATP11C and DOPS (13 ml). Determination of total phospholipid in the samples revealed that the DOPS Nanodisc contains significantly fewer phospholipids (31 phospholipid molecules per Nanodisc) than the others; this is consistent with its apparent smaller size in the SEC analysis. It follows then that empty-Nanodisc produced in the presence of ATP11C and DOPS likely contained both exogenous DOPS and endogenous lipid from the ATP11C sample. What could be expected is that a certain amount of exogenously added DOPS was also distributed in the ATP11C-Nanodisc, and this was proven by the ATPase measurements as described below.

We found that all ATP11C-Nanodiscs were unstable when these samples were stored at a concentration below 1 mg/ml. The amount of ATP11C-Nanodisc detected by SEC analysis was reduced after freeze-thawing or having been stored at 4 °C over an extended period, possibly because the low concentration sample might have bound to the surface of microtubes, resulting in Nanodisc collapse. Collected peak fractions were therefore concentrated to 10 mg/ml and stored at −80 °C until use.

### ATPase activity of ATP11C-Nanodiscs

**Figure 6:**
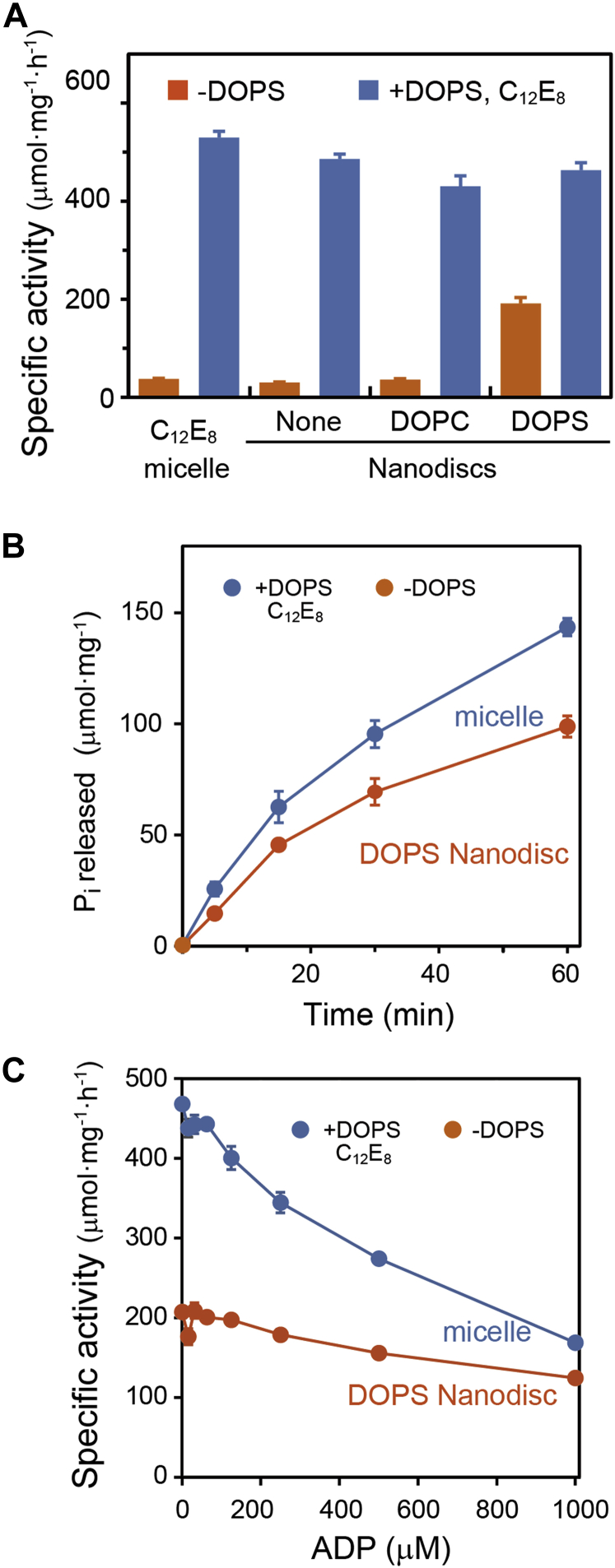
**ATPase activities of ATP11C reconstituted in Nanodiscs.***A*, specific ATPase activities were assayed with or without 100 μM DOPS and 0.03% C_12_E_8_. The data represent mean ± SD obtained from three independent experiments. *B*, time course of P_i_ liberation by ATP11C in DOPS Nanodiscs with or without 100 μM DOPS and C_12_E_8_ was plotted. Data represent the mean ± SEM from triplicate points. *C*, the effect of ADP concentration on the specific activity of ATP11C in DOPS Nanodiscs was examined with or without DOPS and C_12_E_8_. The data represent the mean ± SD.

The function of ATP11C reconstituted in Nanodiscs was analyzed by determining the ATPase activity (Fig. 6). ATP11C is autophosphorylated in the presence of ATP to form an E2P intermediate, which is dephosphorylated upon PtdSer occlusion. We found the release rate of inorganic phosphate (P_i_), detected as ATPase activity, increased with increasing concentrations of PtdSer. The purified sample in detergent micelles, in the absence of DOPS, showed almost no activity, but activity could be measured in the presence of a saturating concentration of 100 μM DOPS dissolved in C_12_E_8_ (*K*_0.5, PS_ = 18.1 μM) (23). The ATP11C-Nanodisc formed with endogenous lipids and that with DOPC exhibited little activity, indicating a negligible amount of PtdSer in these Nanodisc samples. Addition of DOPS and C_12_E_8_, which resolubilized the ATP11C-Nanodisc, increased the ATPase activities of both samples up to the level of the detergent-purified sample, ensuring that the low activities in the absence of DOPS were not due to irreversible denaturation of ATP11C. In contrast, ATP11C-Nanodisc formed with DOPS showed significantly higher ATPase activity relative to that without external addition of DOPS, indicating that DOPS successfully exchanged with endogenous lipids during the reconstitution step. This value without DOPS addition is 41% of that with 100 μM DOPS and 0.03% C_12_E_8_, possibly due to inhibition by a restrictive lipid bilayer or to backward inhibition by an excess of PtdSer in the bilayer as will be discussed. Because ATPase activities were calculated as the specific activity per mg of protein, all the maximum ATPase activities in the Nanodisc samples are slightly underestimated because of the presence of MSP1D1. The turnover numbers per ATP11C calculated for each sample are almost the same for all samples; ATP11C in the detergent micelle: 24.3 s^−1^, ATP11C-Nanodisc with endogenous lipids: 29.0 s^−1^, with DOPC: 25.7 s^−1^, and with DOPS: 27.6 s^−1^, demonstrating that the reconstitution and resolubilization processes did not damage the ATP11C at all.

The time course of activity in the detergent-purified and DOPS membrane-embedded ATP11C-Nanodisc did not show linear P_i_ production over the examined time range (Fig. 5*B*). One possibility could be inhibition caused by ADP production. As expected, the ATPase activities of both samples are inhibited by ADP (Fig. 6*C*). However, contrary to expectations, the sensitivity of detergent and membrane-embedded samples was significantly different: 1 mM ADP reduced the ATPase activity in the detergent micelle to less than 36% of the initial level, whereas the lipid-embedded ATP11C-Nanodisc properties or the E1-E2 equilibrium are different in the membrane *versus* the detergent micelle.

### Structure in the DOPS-Nanodisc

**Figure 7:**
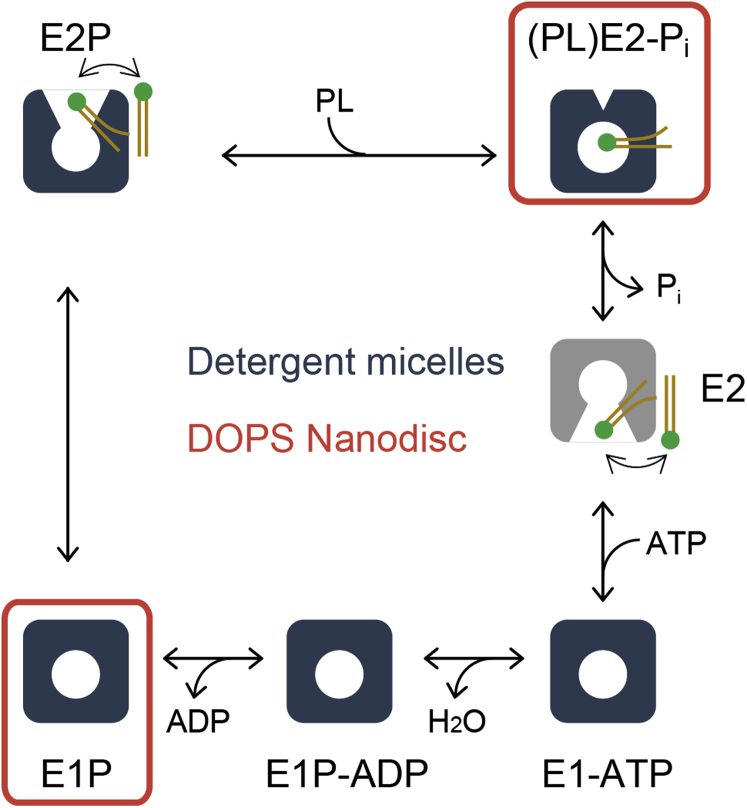
**Post–Albers reaction scheme of PtdSer transport by ATP11C.** A reaction scheme of phospholipid (PL) translocation coupled with ATP hydrolysis is shown. *Cartoons* represent molecular conformations of the ATP11C-CDC50A complex (inward- or outward- and open or occluded states). Intermediate states of the cryo-EM structure of the ATP11C determined in the detergent micelles in our previous study are shown in *blue*, while those determined in this study using ATP11C in the DOPS Nanodisc are indicated as *red boxes*.

**Figure 8:**
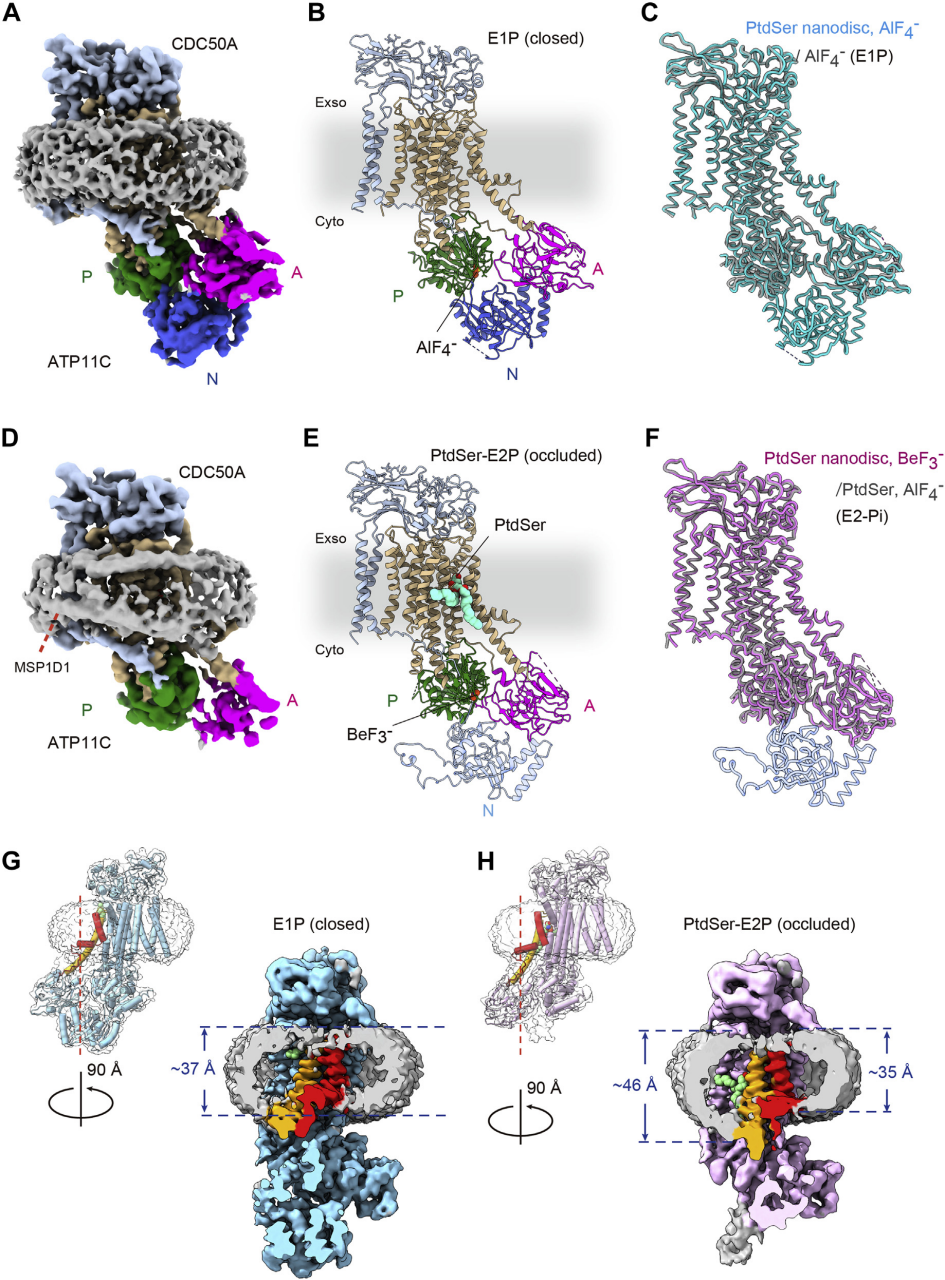
**Cryo-EM structures of ATP11C in DOPS Nanodiscs.** The cryo-EM maps (*A* and *D*) and atomic models (*B* and *E*) of ATP11C in the AlF-bound E1P state (*A* and *B*) and those of BeF-bound (PtdSer)E2-P_i_ state (*D* and *E*), viewed along the membrane plane with exoplasmic-side up. Three cytoplasmic domains (A, P, and N domains). TM helices and accessory subunit CDC50A are shown in different colors, as indicated in the figure. Phosphate analogs (AlF, BeF) bound to the P domain and PtdSer (*pale green*) occluded in the middle of the TM region are shown as *spheres*. EM density for the membrane boundary was segmented and shown as a *gray surface*. *C* and *F*, the line-style model of the E1P (*C*, *cyan*) or (PtdSer)E2-P_i_ state (*F*, *pink*) in the DOPS Nanodisc is superimposed with their corresponding conformations determined in detergent micelles (*gray*) obtained in our previous study. *G* and *H*, slice views of the unmasked EM maps (surface) for E1P (*G*, *blue*) or (PtdSer)E2-P_i_ state (*H*, *pink*) at the clipping position indicated in the figure. TM1 (*red*), TM2 (*orange*), and the discoidal lipid density (*gray*) are highlighted in different colors.

The structure of ATP11C embedded in DOPS Nanodisc was determined by cryo-EM single-particle analysis (Table S1 and Fig. S1). As with the well-studied P2-type ATPases (36, 37, 38), fluorinated phosphate analogs such as aluminum fluoride (AlF) and beryllium fluoride (BeF) were exploited to stabilize discrete reaction intermediates during the transport cycle. We previously reported cryo-EM structures of detergent-purified ATP11C in multiple reaction intermediates, including an outward-open and PtdSer-bound E2P state fixed with BeF, and an outward-facing and PtdSer-occluded E2-P_i_ transition state with AlF (Fig. 7) (22, 23). However, to our surprise, structures for the Nanodisc-embedded ATP11C with AlF and BeF appeared to reflect different reaction intermediates from those obtained in the previous detergent-solubilized samples, as described below (Figs. 7 and 8).

The cryo-EM structure of ATP11C-Nanodisc with AlF was determined at 3.4 Å resolution. In our previous studies using detergent-purified ATP11C, only the E1P state structure was obtained with AlF alone. However, the PtdSer-occluded E2-P_i_ state emerged as the major component (21% of total particles extracted from micrographs), and the E1P state as less prevalent (7.4%) from a single grid prepared in the presence of AlF and DOPS (23). As this Nanodisc sample contained DOPS in its lipid bilayer, this is not unexpected. In contrast, structural features of the ATP11C-Nanodisc with AlF clearly show it to be entirely in the E1P state (Fig. 8, *A*–*C*), and there is no indication of an E2P-like state in the 3D classification step (Fig. S1). The structure of the ATP11C-Nanodisc E1P state is well superimposed onto the detergent-solubilized one (PDB: 7BSQ) with root mean square deviation (RMSD) of 0.428 Å^2^, except for the N domain, which is modeled in the ATP11C-Nanodisc but completely disordered in the detergent sample (Fig. 8*C*). The relatively fixed position of the N-domain in Nanodisc may come about through the bilayer interacting with the nearby A-domain, which, in turn, stabilizes the N-domain *via* the characteristic helix-turn-helix insertion (Fig. 8*A*).

Previously reported BeF-bound crystal and cryo-EM structures of detergent-solubilized ATP11C both represent the outward-open E2P state, in which the extracellular gate is wide open, and PtdSer bound, but not occluded, deep in a longitudinal cleft in the TM region. Unexpectedly, however, the molecular conformation of the BeF-bound ATP11C-Nanodisc formed with DOPS (3.9 Å) is rather close to the outward-facing and PtdSer-occluded E2-P_i_ transition state (Fig. 8, *D*–*F*). The difference between the E2P and E2-P_i_ states is defined by the open/closed state of the extracellular gate and relative orientation of the A and P domains. In the BeF-bound ATP11C-Nanodisc, the exoplasmic gate is closed, and the head group of PtdSer is occluded and thus isolated from the outer leaflet of the membrane. Due to the limited resolution, PtdSer is only partially visible in the EM density map (Fig. S2). However, the binding position of PtdSer and surrounding amino acids especially the extracellular portions of TM1 and TM2, and the accommodation of an acyl chain of PtdSer at the end of TM6 in the ATP11C-Nanodisc sample, are very similar to those observed in the PtdSer-occluded E2-P_i_ state of the detergent-solubilized ATP11C. The overall molecular conformation, including the relative orientation of the A and P domains, is superimposable onto the AlF-bound and PtdSer-occluded E2-P_i_ structure of detergent-solubilized ATP11C (Fig. 8*F*, PDB: 7BSV with RMSD of 0.544 Å^2^). These structural features indicate that, even though we used BeF as the phosphate analog, this BeF-bound form of ATP11C-Nanodisc represents a PtdSer-occluded E2-P_i_ state, which was afforded by AlF in the previous detergent-solubilized sample. In the Nanodisc sample, DOPS is concentrated in the two-dimension membrane plane, so even one DOPS molecule in the inner leaflet of the Nanodisc bilayer, in which roughly 50∼60 phospholipids are present (Table 1), makes its effective concentration high compared with that in the detergent micelle (*K*_0.5, PS_ ∼ 16 μM). We therefore speculate that such a high effective DOPS concentration in the Nanodisc drives the transport cycle one step ahead, from the outward-open E2P to outward-occluded E2-P_i_, even in the presence of BeF as discussed later.

Significantly, besides the detailed molecular event described above, we found a continuous density that surrounds the outermost edge region of the membrane in the PtdSer-E2P state (Fig. 8*D*), which is most likely responsible for the amphipathic α-helical structure of MSP1D1. Such a density was observed neither in detergent micelle samples (23) nor in the E1P state of the Nanodisc sample (Fig. 8*A*). The fact that the density of MSP1D1 is averaged, albeit at a low resolution, indicates that the relative positions of the MSP1D1 belt and reconstituted ATP11C are fixed to some extent by the intermolecular interaction. However, we could not identify any side chains in the TM outermost reach to the MSP1D1 density. It is possible that the interaction is indirect *via* phospholipids at the TM periphery. Although the reason is unclear, these interactions do not occur in the E1P state, thus appear state specific.

It has been proposed that the membrane distortion, or thinning, observed around the transport conduit of phospholipid scramblases likely facilitates the entry/exit of lipid headgroups into the groove and shortens the path for lipid transport (39, 40). Structures of the membrane-embedded ATP11C-Nanodisc allowed us to explore the specific relationship between the membrane layer and protein. We compared the thickness of the lipid-bilayer near TM1–2 and the transport conduit in the two ATP11C-Nanodisc maps (Fig. 8, *G* and *H*). Membrane slices show a significant difference in membrane thickness in the BeF-bound E2-P_i_ form. The membrane around the end of TM1 is restricted in its thickness due to the strongly kinked structure of the cytoplasmic portion of TM1, giving a thickness of approximately 35 Å. In contrast, the membrane around TM2 of the E2-P_i_ state becomes significantly thicker than that around TM1 and other parts of the membrane. The thicker membrane structure observed in the E2-P_i_ form is likely due to the relative orientation of TM2. Basic and amphipathic amino acid residues including Trp111, Arg113, and Arg115 are located at the cytoplasmic portion of TM2 (Fig. S3*A*), which may accommodate phospholipids by “snorkeling” or “floater” as observed in the type-I crystal structures of SERCA (41). As the vertical arrangement of TM2 in the E2-P_i_ state is shifted toward the cytoplasmic side compared with that in the E1P state (Fig. S3*B*), the membrane boundary around the TM2 cytoplasmic side also likely shifts toward the cytoplasmic side along with the TM2 movement during the E1P-E2P transition. The observed protruded membrane boundary structure is consistent with the PtdCho binding pose observed in the corresponding position, near Arg264 and Arg265 in TM2 of Dnf1 in the outward-opened E2P intermediate (Fig. S3*C*) (26). It has been proposed that the exit pathway of the transport phospholipid is through the cytoplasmic portion of TM2 and TM4, based on the structural analysis of ATP8A1 (28), Dnf1 (26) as well as our report for ATP11C (23). In contrast to the membrane thinning observed in scramblases, the protruded membrane structure around the phospholipid exit of ATP11C flippase suggests that the phospholipid poised to be flipped to the inner leaflet is momentarily located at this position along with TM2 and then released smoothly to the inner leaflet of the bilayer. Our structures of ATP11C embedded in the lipid membrane, analogous to authentic E1P and E2-P_i_, indicate that the membrane boundary varies and is no longer flat around the protein in E2-P_i_, a change that likely contributes to phospholipid translocation across the membrane leaflets.

## Discussion

Detergent removal using polystyrene beads such as Biobeads is well established for Nanodisc reconstruction of membrane proteins (27, 29, 30, 38, 42). The yield of the Nanodisc assembly by this procedure has been reported to range from 10% to 90% (31). In some cases, due to the hydrophobic nature of the surface of polystyrene beads, membrane proteins are nonspecifically attached to the surface of the beads, which affects the efficiency and reproducibility of Nanodisc assembly. On the other hand, MβCD accommodates a detergent molecule in the hydrophobic pocket formed inside its cyclic carbohydrate structure, but absorption of membrane proteins here is unlikely considering their size (43).

We found that the temperature during detergent removal is a crucial factor in Nanodisc formation (Fig. 2). A previous report on Nanodisc reconstitution has suggested that Nanodisc assembly progresses as long as the temperature is above the phase transition temperature of the phospholipids used (−22 °C and −10 °C for DOPC and DOPS, respectively) because then these phospholipids behave as a liquid (44). This led us to expect that a relatively low temperature (*e.g.*, 4 °C or on ice) would be needed. Instead, we found that a temperature of more than 30 °C significantly increases the yield of Nanodiscs (Fig. 2*E*). This may be attributed to phospholipid exchange kinetics, similar to the temperature-dependent exchange of phospholipids between detergent-solubilized SERCA and phospholipid-detergent mixed micelles (45).

We also found that the molar ratio of MSP1D1 to C_12_E_8_, rather than MβCD to C_12_E_8_, is a critical factor in Nanodisc assembly (Figs. 3 and 4). MSP1D1 adheres to lipids as well as detergent. Where the proportion of MSP1D1 is insufficient to completely cover the detergent-lipid-mixed micelles MβCD could strip the detergent directly from the mixed micelles surrounding the membrane protein, resulting in protein aggregation and hence a low yield of products. This may be a reason why excess MβCD does not completely recover the yield of ATP11C-Nanodisc formation in the presence of a high concentration of C_12_E_8_ (Fig. 4*C*).

In this study, we employed the molar ratio of MβCD to C_12_E_8_ = 1:1 for preparative scale ATP11C-Nanodisc assembly. We were concerned about the undesirable effect of excess MβCD, because of how excess MβCD extracted phospholipids from rod outer-segment disk membranes (46). More recently, MβCD has been used to remove phospholipid from Nanodiscs in the analysis of a mechanosensitive ion channel, MscS (47).

Determination of the total amount of phospholipids in the samples revealed that an unexpectedly large amount (92 phospholipid molecules per ATP11C) was included in the affinity-purified ATP11C sample. Since these phospholipids are not removed by the washing step during affinity purification with C_12_E_8_-containing buffer, they must be rather strongly bound to the hydrophobic surface of the TM region of ATP11C, and indeed some of them are visualized as cylindrical densities in the detergent-solubilized ATP11C structure (23). However, as the addition of DOPS and C_12_E_8_ induces ATPase activation, these phospholipids seem to be exchangeable with externally added phospholipids.

The question then arises as to how many phospholipids are exchanged upon Nanodisc formation? When we prepared ATP11C-Nanodisc in the presence of DOPS, a molar ratio of ATP11C:endogenous phospholipid:DOPS = 1:92:33 is expected in the system. If we assume a free exchange of endogenous phospholipids and exogenous DOPS in the ATP11C-Nanodisc, approximately a quarter of the phospholipids should have been substituted for DOPS. Since only 50∼60 phospholipids are distributed in the two-dimension lipid bilayer formed in the restricted space of the ATP11C-Nanodisc, the effective concentration of DOPS is extremely high. Our previous structural analysis (23) showed that when PtdSer is loosely bound to the TM binding site in the outward-open E2P state, a few hydrogen bonds are formed between its head group and surrounding amino acids. However, in the subsequent outward-occluded (PtdSer)E2-P_i_ state, PtdSer head group is tightly coordinated with many hydrogen bonds in the TM-binding site. This includes the hydrogen bond formed between PtdSer head group and the extracellular portions of TM1 and 2, which is likely to be a driving force for extracellular gate closure, thus propelling the transport cycle forward. Because TM1 and 2 connect to the A domain, their movement upon hydrogen bond formation with the PtdSer head group is likely coupled to the A domain shift that occurs during E2P to E2-P_i_, even in the presence of bound BeF. Therefore, the presence of a high effective concentration of PtdSer in the Nanodisc bilayer may lead to a high occupancy of the loosely bound PtdSer at the TM-binding site in the outward-open E2P-like state, and we speculate that this PtdSer eventually interacts with the extracellular gate (extracellular portion of TM1 and 2), which comes to the site by thermal fluctuation, and fixes the PtdSer in a more stable occluded state. This may be the reason for the unexpected PtdSer-occluded E2-P_i_ state-like conformation observed in the BeF-bound form of the ATP11C-Nanodisc.

Several cases of the gate-closed E2BeF conformation have been reported in other P-type ATPases; in P4-type, the E2BeF state of ATP8A1 (28) and the autoinhibited form of Drs2p (24) show a closed extracellular gate. In the case of P2-type H^+^,K^+^-ATPase, we have previously demonstrated that BeF alone forms a structure similar to that of the luminal-closed E2-P_i_ state in its two-dimensional crystal structure (48), and this is fixed to the luminal-open state in the presence of its specific inhibitor that binds near the luminal gate (20). In the same P2-type SERCA, the gate is clearly open only in the crystal structure formed in the presence of a high concentration of Mg^2+^ (37), and the gate does not completely open in the presence of BeF alone or with thapsigargin (38). A similar structure of the E2BeF state has recently been reported for the polyamine-transporting P5B-ATPase as well (49). The above examples support the notion that the balance of forces required for TM gate-closure is isoform specific, and consequently, the obtained conformation need not be the outward-open form even using BeF as a phosphate analog.

In our previous crystal structure of the BeF-bound E2P state of ATP11C, we identified a putative bound PtdSer at the extracellular cavity formed by the TM3–4 loop and CDC50A (22). This binding site has come under scrutiny because an unusually high concentration of DOPS (>10 mM) and C_12_E_8_ were present in the crystal solution. Since the present ATP11C-Nanodisc is embedded in the membrane at a high DOPS concentration, we expect, if an extracellular PtdSer-binding site exists physiologically, that it would be in the present EM structure. However, in both ATP11C-Nanodisc structures in AlF-bound E1P and BeF-bound E2-P_i_ states, there is no indication of such PtdSer binding at the extracellular site. It seems to only appear at a higher concentration of DOPS and detergent, and its physiological relevance remains to be ascertained.

## Conclusion

Here we revisited the procedure for Nanodisc formation by MβCD. After optimization of several parameters, including molar ratios of reagents and incubation temperature, ATP11C was reconstituted into Nanodiscs with high efficiency and quality sufficient for structural and functional studies. A cryo-EM structure of ATP11C embedded in the lipid bilayer revealed a significant membrane protrusion in the vicinity of the end of TM2. This could be the first sighting of the elusive phospholipid release site to the inner leaflet.

## Experimental procedures

### Protein expression and purification

The procedures for protein production were as described with some modification (22). Human ATP11C (NCBI: XM_005262405.1) with a 7 aa truncation at the N terminus and 38 aa at the C terminus (ATP11X_cryst) was tagged with FLAG-Hisx6-EGFP-TEV cleavage site at the N terminus. The FLAG-Hisx6-EGFP-TEV-ATP11C and human CDC50A (NCBI: NM_018247.3) were coexpressed in Expi293 cells (Thermo) using a baculovirus-mediated transduction system (50, 51). The harvested cells were solubilized in 1.5% (w/v) n-decyl β-D-maltoside in a lysis buffer containing 40 mM MES/Tris (pH 6.5), 200 mM NaCl, 2 mM MgCl_2_, 1 mM ATP, 1 mM dithiothreitol, 0.1 mM ethylene glycol-bis(2-aminoethylether)-N,N,N′,N′-tetraacetic acid (EGTA), and protease inhibitor cocktail (Roche) for 30 min at 4 °C. The solubilized fraction was centrifuged at 200,000*g* for 1 h. The supernatant was collected, mixed with GFP-binding nanobody resin (52, 53), and incubated for 1 h at 4 °C. The resin was washed with a buffer containing 20 mM MES/Tris (pH 6.5), 200 mM NaCl, 5% (v/v) glycerol, 1 mM MgCl_2_, 0.1 mM ATP, 0.1 mM EGTA and 0.03% octaethylene glycol monododecyl ether (C_12_E_8_, Nikko Chemical). TEV protease and EndoHf (New England Biolabs) were added to the suspension. After protein digestion overnight at 4 °C, proteins released from the GFP-binding nanobody were collected. The protease and the endoglycosidase therein were absorbed by a Ni-NTA resin (QIAGEN). The nonabsorbed fractions were concentrated to 4 to 5 mg/ml and kept at −80 °C before use.

The pMSP1D1 plasmid was purchased from Addgene (#20061), transformed into *E. coli* BL21-Gold (DE3). The MSP1D1 protein was expressed, purified from the culture as described. The purified MSP1D1 was dialyzed against 10 mM Tris-HCl, pH 8.0, 0.1 M NaCl, 0.5 mM EDTA, concentrated to 10 mg/ml, and kept at −80 °C before use.

### Empty Nanodisc assembly using methyl-β-cyclodextrin

Lipid Nanodiscs without membrane protein (empty Nanodisc) were generated with scaffold protein MSP1D1 and phospholipids. DOPC and DOPS (Avanti) dissolved in chloroform were dried under a N_2_ gas stream, solubilized to 10 mg/ml in 2% C_12_E_8_, and stored at −20 °C. MβCD was dissolved in Milli-Q water and stored at −20 °C. Each reaction contained 20 μM MSP1D1, 0 to 1.3 mM phospholipids in 100 μl of the SEC buffer consisting of 20 mM MES/Tris (pH 6.5), 50 mM NaCl, 5 mM MgCl_2_. A Voltex mixer was used to mix the solution immediately after adding MβCD and incubated for 10 min at 30 °C. The aggregates were removed through a 0.22 μm filter using centrifugal units or precipitated by centrifugation at 50,000 rpm for 20 min at 4 °C. The samples (40 μl each) were subjected to analytical SEC using Superdex200 Increase 5/150 GL and the SEC buffer. The separation profiles were detected by absorbance at 280 nm on the HPLC system (Shimadzu). The intensity of tryptophan fluorescence (excitation 280 nm, emission 320 nm) derived from MSP1D1 fluctuated widely depending on temperature, detergent, and other factors, and failed to adequately evaluate Nanodisc assembly.

### ATP11C-CDC50A Nanodisc assembly

The ATP11C-CDC50A Nanodisc (ATP11C-Nanodisc) was assembled with ATP11C, MSP1D1, and phospholipids. The affinity-purified and concentrated ATP11C-CDC50A sample retained roughly 0.3 to 0.5% C_12_E_8_. Each incubation sample contained 10 μM ATP11C-CDC50A complex, 20 to 160 μM MSP1D1, and 0 to 1.3 mM phospholipids in 100 μl of the SEC buffer. The Nanodisc was assembled by adding MβCD and separated by SEC as described above. Protein concentrations were determined by the calculated molecular extinction coefficient at 280 nm.

### Large scale preparation of ATP11C-CDC50A Nanodiscs

The ATP11C Nanodisc assembly was scaled-up for structural analysis. Each reaction contained 14 μM (2.3 mg) ATP11C-CDC50A, phospholipids, MSP1D1, and 0.3 to 0.5% C_12_E_8_ carried over from the protein and the phospholipids. The molar ratios of ATP11C-CDC50A:lipid:MSP1D1 were set to 1:0:8 in ATP11C-endogenous lipid Nanodisc, 1:65:8 in ATP11C-DOPC Nanodisc, and 1:65:16 in ATP11C-DOPS Nanodisc, respectively. Their assembly was initiated by adding an aliquot of 100 mM MβCD solution at the molar ratio MβCD:C_12_E_8_ of 1:1. The sample was preincubated at 30 °C for 5 min, immediately mixed after adding MβCD, then incubated at 30 °C for 15 min. After centrifugation at 50,000 rpm for 20 min at 4 °C, the supernatant was separated by SEC using Superdex 200 Increase 10/300 GL with the SEC buffer at 4 °C. The peak fractions were collected, concentrated to 10 mg/ml. The aliquots were flash-frozen and stored at −80 °C until use.

### ATPase assay

The purified ATP11C-Nanodiscs were subjected to an ATPase activity assay as described (54). Briefly, The ATP11C-Nanodisc preparation was diluted in SEC buffer with or without 100 μM DOPS and 0.03% C_12_E_8_, and the aliquots were mixed with an assay buffer comprising 20 mM MES/Tris (pH 6.5), 100 mM NaCl, 2% glycerol, 2 mM MgCl_2_, 2 mM ATP, 0.03 mg/ml of C_12_E_8_ (pH 7.0 adjusted by Tris) with or without both 100 μM DOPS and 0.03% C_12_E_8_, in 96-well plates. The reaction with 1 mM BeSO_4_ and 3 mM NaF (BeF_3_^−^) served as a blank. The 96-well plates were incubated for 30 min at 37 °C using a thermal cycler. The reactions were stopped by adding an equal volume of 10% SDS. The amount of released P_i_ was determined colorimetrically using a microplate reader (TECAN).

### Determination of total phosphorus

Total phosphorus derived from the phospholipids in the Nanodiscs was quantified by the molybdate method (55). Microgram amounts (range 10–300 μg) of ATP11C-Nanodisc, affinity-purified ATP11C, and empty-Nanodisc were subjected to the determination.

### Grid preparation for electron microscopy

Cryo-EM grids were prepared as described (28) with some modifications. The concentrated ATP11C-DOPS Nanodisc preparation (10 mg/ml) was preincubated either with 1 mM BeSO_4_ and 3 mM NaF (BeF_3_^−^) or with 1 mM AlCl_3_ and 4 mM NaF (AlF_4_^−^) for 30 min on ice. Fos-Choline-8 fluorinated was added just before grid preparation. Three microliters of the 7.5 mg/ml sample containing 0.05% Fos-Choline-8 fluorinated was applied to a freshly glow-discharged Quantifoil holey carbon grid (R1.2/1.3, Cu/Rh, 300 mesh), blotted under 99% humidity condition using a Vitrobot Mark IV (FEI) set at force 10 for 8 s at 4 °C, and then the grids were plunge-frozen in liquid ethane.

### Electron microscopy data collection and processing

The prepared grids were transferred to a Titan Krios G3i microscope (Thermo Fisher Scientific), running at 300 kV and equipped with a Gatan Quantum-LS Energy Filter (GIF) and a Gatan K3 Summit direct electron detector in the electron counting mode. Imaging was performed at a nominal magnification of 105,000×, corresponding to a calibrated pixel size of 0.83 Å/pix (The University of Tokyo). Each movie was recorded for 3.2 s and subdivided into 54 frames. The electron flux was set to 14 e^−^/pix/s at the detector, resulting in an accumulated exposure of 64 e^−^/Å^2^ at the specimen. The data were automatically acquired by the image shift method using SerialEM software (56), with a defocus range of −0.8 to −1.6 μm. Approximately 3000 movies were acquired for each condition, and the numbers of total images are described in Table S1. For all datasets, the dose-fractionated movies were subjected to beam-induced motion correction, using MotionCor2 (57) or Relion (58), and the contract transfer function (CTF) parameters were estimated using CTFFIND4 (59).

For each dataset, particles were initially picked using the Laplacian-of-Gaussian picking function in RELION-3 (58) and extracted with downsampling to a pixel size of 3.24 Å/pix. These particles were subjected to several rounds of 2D and 3D classifications. The best class was then reextracted with a pixel size of 0.83 Å/pix and subjected to 3D refinement. The resulting 3D model and particle set were subjected to per-particle defocus refinement, beam-tilt refinement, Bayesian polishing (60), and 3D refinement. The resolution of the analyzed map is defined according to the FCS = 0.143 criterion (61). The local resolution and angular distributions for each structure were estimated by Relion.

### Model building and validation

All the models were manually built in COOT (62) using cryo-EM structures of the ATP11C of which the PDB codes were 7BSS (E1P), 7BSV (E2-P_i_) as starting models. After adjustment of the model into the EM density map, structure refinement was performed with Phenix (63). The N domain in E2-P_i_ states was not modeled due to the sparse densities for the corresponding part in the EM density maps. We instead showed the N domain derived from the ATP11C crystal structure (22) by superimposing the crystal structure to the cryo-EM model to show the approximate position of the N domain in the E2-P_i_ state (Fig. 8, *E* and *F*). The statistics of the 3D reconstruction and model refinement are summarized in Table S1. All molecular graphics were prepared using UCSF Chimera (64), UCSF Chimera X (65), and PYMOL (https://pymol.org).

## Data availability

The data that support this study are available from the corresponding author upon reasonable request. The data needed to evaluate the conclusion of the paper are either in the paper or the Supporting Information. Following atomic models and cryo-EM maps have been deposited in PDB (https://www.rcsb.org) and Electron Microscopy Data Bank, respectively. 7VSG and EMD-32110: Cryo-EM structure of a human ATP11C-CDC50A flippase reconstituted in the Nanodisc in PtdSer-occluded E2-Pi state. 7VSH and EMD-32111: Cryo-EM structure of a human ATP11C-CDC50A flippase reconstituted in the Nanodisc in E1P state.

## Supporting information

This article contains supporting information.

## Conflict of interest

The authors declare that they have no conflicts of interest with the contents of this article.
