# Supplementary material for: Cryo-EM of the ATP11C flippase reconstituted in Nanodiscs shows a distended phospholipid bilayer inner membrane around transmembrane helix 2
Source: J Biol Chem. 2021 Dec 17;298(1):101498. doi: 10.1016/j.jbc.2021.101498 (PMC8733269; doi:10.1016/j.jbc.2021.101498)
Supplement: Supplemental Figures S1–S3 and Table S1 [file mmc1.pdf]

Cryo-EM of the ATP11C flippase reconstituted in Nanodiscs shows a distended phospholipid bilayer inner membrane around transmembrane helix 2

Hanayo Nakanishi[1], Kenichi Hayashida[1], Tomohiro Nishizawa[2], Atsunori Oshima[1,3,4] & *Kazuhiro Abe[1,3]

[1]Cellular and Structural Physiology Institute, Nagoya University, 464-8601, Japan

[2]Graduate School of Medical Life Science, Yokohama City University, Tsurumi, Yokohama, 230-0045, Japan

[3]Graduate School of Pharmaceutical Sciences, Nagoya University, 464-8601, Japan

[4]Institute for Glyco-core Research (iGCORE), Nagoya University, Furo-cho, Chikusa-ku, Nagoya 464-8601, Japan

*Correspondence: Kazuhiro Abe, kabe@cespi.nagoya-u.ac.jp

Supporting information

Table S1| Cryo-EM data collection, processing, refinement and validations

	AlF (E1P)<br>(EMD-32111)<br>(PDB 7VSH)	BeF (PtdSer-occluded $E2P_i$)<br>(EMD-32110)<br>(PDB 7VSG)
**Data collection and processing**		
Magnification	105,000	
Voltage (kV)	300	
Electron exposure (e–/Å^2)	48	
Defocus range (μm)	0.8 – 1.8	
Pixel size (Å)	0.83	
Symmetry imposed	*C1*	
Initial particle images (no.)	524,504	1,038,183
Final particle images (no.)	78,800	143,384
Map resolution (Å)	3.4	3.9
FSC threshold	0.143	0.143
**Refinement**		
Initial model used (PDB code)	7BSQ	7BSV
Model resolution (Å)	3.4	3.8
FSC threshold	0.5	0.5
Map sharpening *B* factor (Å^2)	-93.1	-141.0
Model composition		
Non-hydrogen atoms	11001	9333
Protein residues	1352	1139
Ligands	1 MAN, 4 NAG, 1 MG, 1 ALF, 1 17F	1 MAN, 4 NAG, 1 17F
B factors (Å^2)		
Protein	34.32	53.54
Ligand	38.11	58.51
R.m.s. deviations		
Bond lengths (Å)	0.004	0.003
Bond angles (°)	0.641	0.673
Validation		
MolProbity score	1.79	1.72
Clashscore	6.88	9.49
Poor rotamers (%)	0.25	0.10
Ramachandran plot		
Favored (%)	93.9	96.6
Allowed (%)	6.11	3.37
Disallowed (%)	0	0

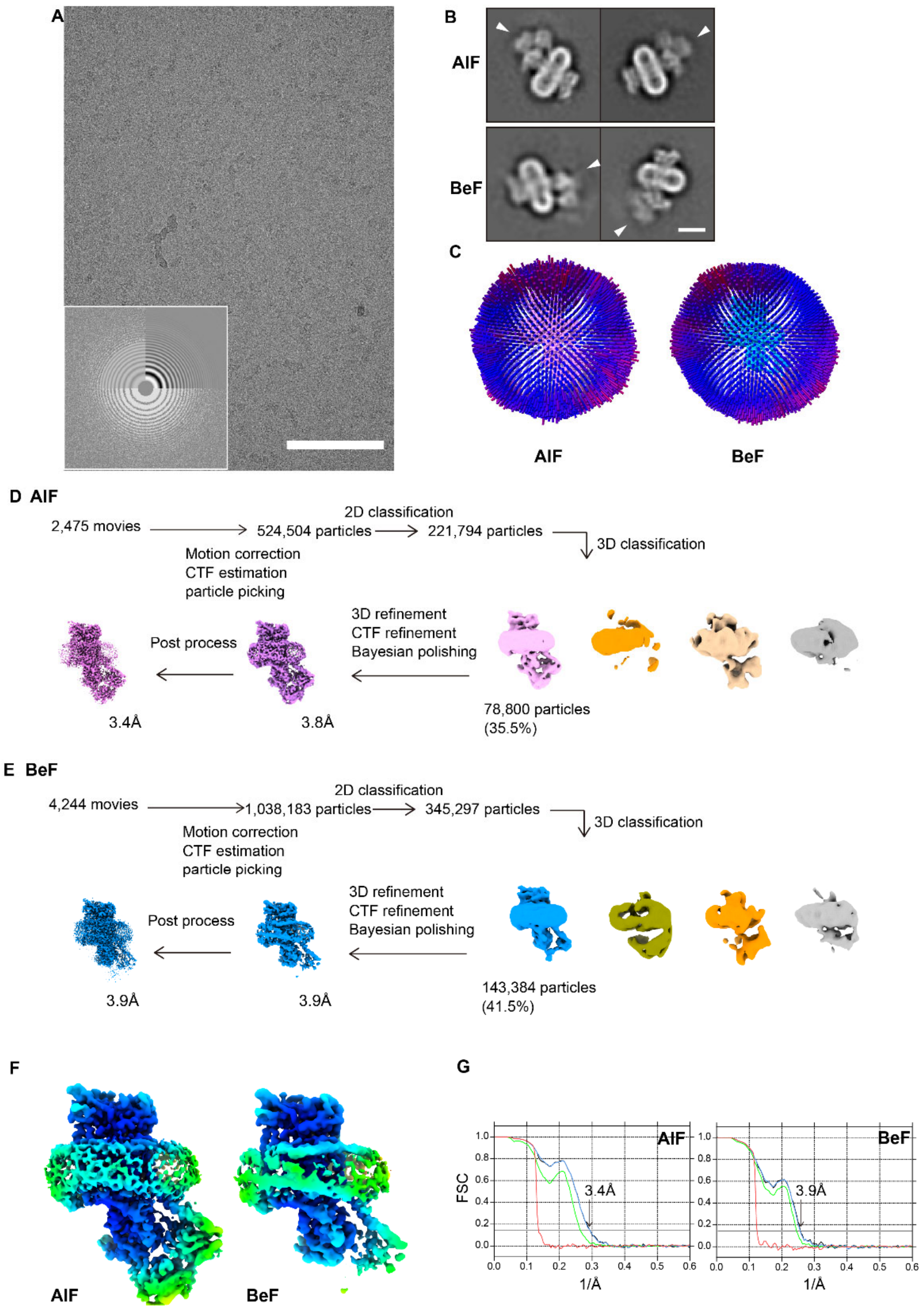

Figure S1. Cryo-EM analysis of ATP11C-nanodisc

(A) A representative cryo-EM image and its Fourier transform. Bar, 50 nm. (B) Representative 2D-class averages. Bar, 5 nm. Arrowheads indicate the N domain in each sample, which is disordered in the BeF form. (C) Angular distribution plot of particles included in the final 3D reconstruction. The number of views at each angular orientation is represented by the length and color of cylinders. Red indicates more views. (D,E) Data processing (see Methods). (F) Final reconstruction map colored by local resolution as calculated by RELION3.1. (G) FSC plot used for resolution estimations (black: corrected, blue: masked, green: unmasked, red: phase randomized).

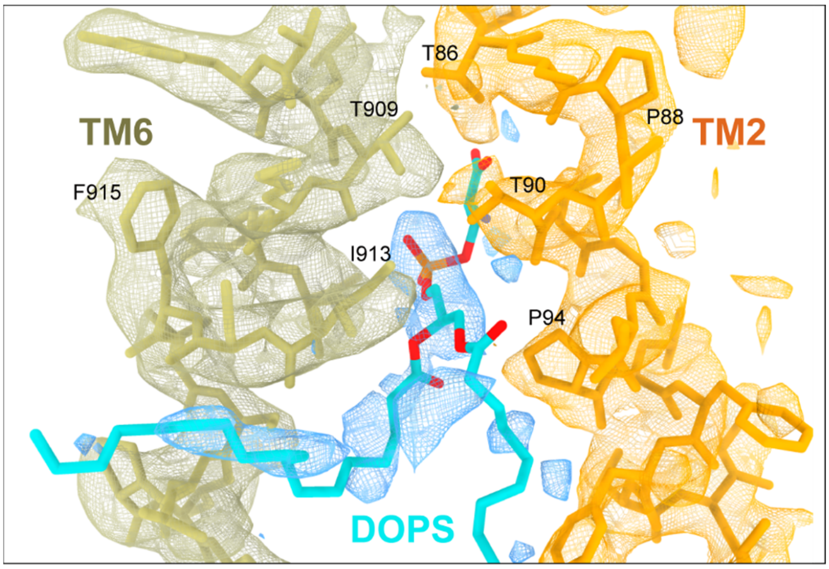

Figure S2. Cryo-EM density for the occluded DOPS in the transport groove.
Mesh represents cryo-EM density maps around the bound DOPS (cyan), TM2 (tangerine), and TM6 (tan) of the ATP11C in the E2P state. Some of the key residues around them are indicated. Contour levels were set at 2.2 σ.

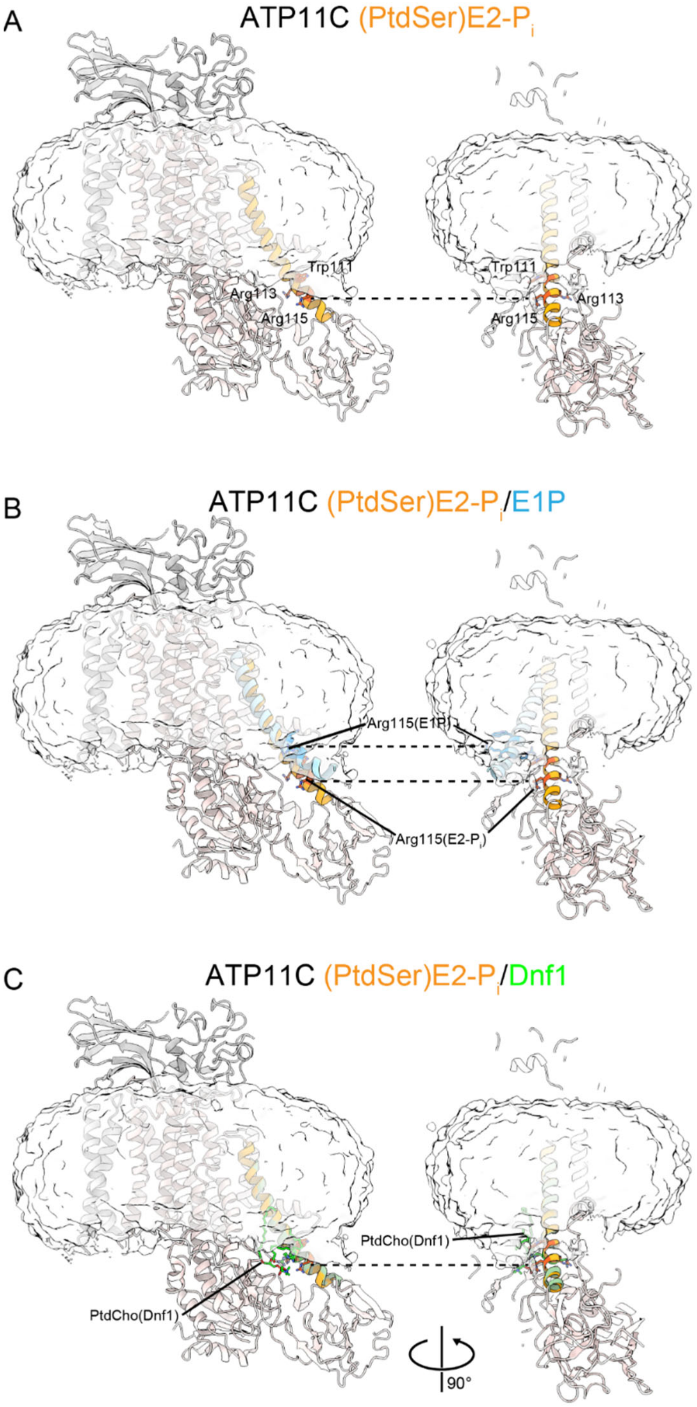

Figure S3. Displacement of the membrane boundary along with TM2.
(A) The membrane protrusion around the TM2 cytoplasmic side. Basic and amphipathic amino acid residues on the cytoplasmic portion of TM2 are indicated. (B) The position of Arg115 on TM2 was compared between the (PtdSer)E2-P_i state and the E1P state. (C) The position of PtdCho binding to TM2 of Dnf1-Lem3 in the outward-opened E2P state was compared with the membrane protrusion of ATP11C in the (PtdSer)E2-Pi state. The figure shows the side and front views of the discoidal lipid density in the unmasked EM maps (translucent surface) and the atomic models (pale pink). The TM2 of ATP11C and Dnf1-Lem3 are highlighted in different colors.
